# Effects of Biochar on Soil Microbial Biomass after Four Years of Consecutive Application in the North China Plain

**DOI:** 10.1371/journal.pone.0102062

**Published:** 2014-07-15

**Authors:** Qing-zhong Zhang, Feike A. Dijkstra, Xing-ren Liu, Yi-ding Wang, Jian Huang, Ning Lu

**Affiliations:** 1 Key Laboratory of Agricultural Environment, Ministry of Agriculture, Sino-Australian Joint Laboratory For Sustainable Agro-Ecosystems, Institute of Environment and Sustainable Development in Agriculture, Chinese Academy of Agricultural Sciences, Beijing, China; 2 Centre for Carbon, Water and Food, Department of Environmental Sciences, The University of Sydney, Camden, New South Wales, Australia; NERC Centre for Ecology & Hydrology, United Kingdom

## Abstract

The long term effect of biochar application on soil microbial biomass is not well understood. We measured soil microbial biomass carbon (MBC) and nitrogen (MBN) in a field experiment during a winter wheat growing season after four consecutive years of no (CK), 4.5 (B4.5) and 9.0 t biochar ha^−1^ yr^−1^ (B9.0) applied. For comparison, a treatment with wheat straw residue incorporation (SR) was also included. Results showed that biochar application increased soil MBC significantly compared to the CK treatment, and that the effect size increased with biochar application rate. The B9.0 treatment showed the same effect on MBC as the SR treatment. Treatments effects on soil MBN were less strong than for MBC. The microbial biomass C∶N ratio was significantly increased by biochar. Biochar might decrease the fraction of biomass N mineralized (*K*_N_), which would make the soil MBN for biochar treatments underestimated, and microbial biomass C∶N ratios overestimated. Seasonal fluctuation in MBC was less for biochar amended soils than for CK and SR treatments, suggesting that biochar induced a less extreme environment for microorganisms throughout the season. There was a significant positive correlation between MBC and soil water content (SWC), but there was no significant correlation between MBC and soil temperature. Biochar amendments may therefore reduce temporal variability in environmental conditions for microbial growth in this system thereby reducing temporal fluctuations in C and N dynamics.

## Introduction

Biochar is the product of the thermal degradation of organic materials in the absence of air (pyrolysis) and is distinguished from charcoal by its use as a soil amendment [1]. Application of biochar has been proposed as a novel approach to improve soil fertility, increase soil carbon sequestration and mitigate the greenhouse effect [2]. Nevertheless, the turnover of soil organic matter and biochar and their interaction in soils remain poorly understood. Positive and negative priming effects on native organic carbon mineralization in biochar-amended soils have been reported [3]–[6], and the priming direction is thought to be controlled by the biochar type (determined by production conditions and sources used) and soil pH. Moreover, the presence of soil organic matter can stimulate the mineralization of the more labile components of biochar over the short term, but over the long term, the biochar-soil interaction may enhance soil C storage via processes of organic matter sorption to biochar and physical protection [3], [5], [6]. There is strong evidence that priming effect on soil organic matter decomposition relies on microbial biomass [7]. Research on soil microbial biomass dynamics with biochar application will help the understanding of priming effect on soil organic matter and biochar decomposition. Biochar addition can increase soil microbial biomass, and may also affect the soil biological community composition, which in turn will affect nutrient cycling, plant growth, and greenhouse gas emission, as well as soil organic carbon mineralization mentioned above [1].

However, the present knowledge on soil microbial biomass dynamics due to biochar application is mainly based on the comparison between biochar application and no biochar treatment without considering crop residue return practice, and lacks long-term field experimental data [8]–[12]. Our field experiment with consecutive biochar amendments and crop residue return lasted nearly 4 years in a winter wheat-maize relay cropping system until October 2010, with total inputs of biochar ranging from 18.0 to 36.0 t ha^−1^. The aim of this paper is to examine effects of biochar and wheat straw residue incorporation on the temporal and spatial variation in microbial biomass carbon (MBC) and microbial biomass nitrogen (MBN) measured within a wheat growing season under different soil depths after four years of consecutive application.

## Materials and Methods

### Site description

The field experiment was conducted at an experimental station (36°58′N, 117°59′E, elevation 17 m) for ecological and sustainability research in Huantai County, Shandong Province, China, and was begun in 2007 [13]. This site has a warm, temperate, continental monsoon climate with a mean annual temperature of 12.4°C. The mean annual precipitation was 600 mm, with most rainfall occurring in June, July, and August. The soil is classified to Fluvic Cambisol according to the USDA system. The soil was a sandy loam, and the proportion of sand, silt, and clay particles in the top 20 cm of soil was 70.8, 26.9, and 2.3%, respectively. The soil bulk density of the top 20 cm of soil before the biochar amendment was 1.52 g cm^−3^, and the soil organic matter (SOM) content was 15 g kg^−1^ of soil. The soil pH was 8.1 before experiment, and was not significantly changed due to biochar amendment [13].

### Biochar

Milled biochar was purchased from a local company (Jinfu Biochar Company, Liaoning Province), with a density of 0.297 g cm^−3^ and a pH of 8.2 [14]. The biochar was made by incomplete self-combustion of crushed corncob in an open-top concrete tank with an igniting apparatus at the bottom for about 24 hours at about 360°C. The incomplete self-combustion process did not need an external energy source. The biochar contained 65.7% carbon and 0.909% N (analyzed with an elemental analysis apparatus, Flash EA 2000, Thermo Electron Corporation, Italy). Available phosphorus content was 0.08% (extracted with 0.5 M NaHCO_3_ at a pH of 8.5, and analyzed with a colorimetric method), and available potassium content was 1.60% (extracted with 2.0 M HNO_3_, and analyzed with a flare photometer, FP640, Cany, China). Ash content of the biochar was 72.0% (determined by dry combustion in a muffle furnace at 550°C for 2 h).

### Experimental design

The experimental crops were winter wheat (*Triticum aestivum* L.) and maize (*Zea mays* L.) in relay cropping. Generally, the maize was sown in early June and matured in late September. The winter wheat was then sown in early October and harvested in early June of the next year. The field experiment was a randomized complete block design and each of the experimental plots was 6 m×6 m = 36 m^2^, four treatments (CK, B4.5, B9.0, SR) with three replications. The experiment included a control treatment with no biochar addition (CK), two biochar treatments with 4.5 and 9.0 t ha^−1^ yr^−1^ biochar applied (B4.5 and B9.0, respectively), and a treatment where all wheat and maize straw residue produced in the plot was returned to the field and incorporated into the soil after harvest by a straw returning machine (SR), which allowed us to compare biochar vs. fresh litter effects on microbial biomass. Based on the estimated total amount of aboveground crop residues of about 15 t ha^−1^ yr^−1^ for local normal croplands and the empirical value of about 30% in weight of crop residues left as biochar from the local biochar company, biochar amount can be obtained from the field crop residues is equivalent to that used in the B4.5 treatment.

The biochar was distributed equally to each crop (half applied before sowing of wheat and the other half before sowing of maize). Inorganic basal N fertilizers as urea of 200 kg N ha^−1^ yr^−1^ (165 kg N ha^−1^ yr^−1^ was used before 2009), P as superphosphate of 52.5 kg P_2_O_5_ ha^−1^ yr^−1^, and K as potassium sulfate of 37.5 kg K_2_O ha^−1^ yr^−1^ were applied in all treatments. Before 2009, all fertilizers were used as base fertilizer. From 2009, half of nitrogen fertilizer was applied as base fertilizer, and the other half was applied as topdressing. Biochar and the base fertilizers were broadcast on the soil surface and incorporated into the soil by rotary tillage to depth of 15 cm before seeding.

### Soil sampling

Soil samples were collected during the winter wheat growing period (from October 2010 to June 2011) divided into five functional stages: (1) wheat post-seeding stage; (2) before freezing stage, (3) reviving stage; (4) shooting stage, and (5) harvest stage. Soil samples were collected at depths of 0–5, 5–10, 10–20, and 20–30 cm in each experimental plot. The soil samples were collected from five points randomly, and mixed into one sample, each mixed soil sample was divided into two parts. One part of the soil sample was determined for soil water content (SWC) and the other part was prepared for microbial analysis. All samples were immediately stored in sealed plastic bags in a cooler and transported to laboratory and stored in refrigerator at 4°C. All microbiological determinations were performed within one week of sampling.

### Measurement and monitoring

Microbial biomass carbon (MBC) and nitrogen (MBN) in soil were determined by fumigation extraction method [15], [16], and the value of 0.45 was used for both the fraction of biomass C mineralized (*K*_C_) and the fraction of biomass N mineralized (*K*_N_). Soil samples were thoroughly mixed and ground to pass through a 2-mm sieve, and then the soil moisture was adjusted to about 40% water holding capacity. We fumigated 20.0 g (dry weight equivalent) of soil with ethanol-free chloroform for 24 h. Both fumigated and non-fumigated soils were extracted with 80 ml of 0.5 M K_2_SO_4_ by shaking for 30 min on a reciprocating shaker at 40 cycles per minute and then filtered (soil∶water = 1∶4). The TOC analyzer (Multi N/C 2100, Jena, Germany) was used to determine the C and N in the extracts.

SWC was determined gravimetrically by oven-drying at 105°C for 48 h. Soil temperature at 5-cm depth in each replication was monitored hourly by a temperature probe (tolerance: ±0.2°C over the 0–70°C range; temperature measurement range: −50 to +70°C; model 109, Campbell Scientific, Logan, UT, USA) connected to a datalogger (model U12-006, Jimuduoli, Beijing, China) from 22 October 2010, except during harvesting and sowing (June 10–July 2) of each year.

### Data analyses

We used repeated measures ANOVA to test for main effects of treatment (between-subjects factor), soil depth (within-subjects factor) and date (within-subjects factor), and their interactive effects on MBC, MBN and microbial biomass C∶N ratio. When necessary, data were log-transformed to reduce heteroscedasticity and improve assumptions of normality. We used Pearson's test to determine whether there was a significant correlation between microbial and environmental soil properties (moisture and temperature) and Fisher's least significant difference (LSD) test to determine the significant difference in coefficient of seasonal variability (CV) of MBC and MBN between different treatments. All statistical analyses were performed with JMP (version 4.0.4; SAS Institute, Cary, NC, USA).

## Results

### Microbial biomass C (MBC)

**Figure 1:**
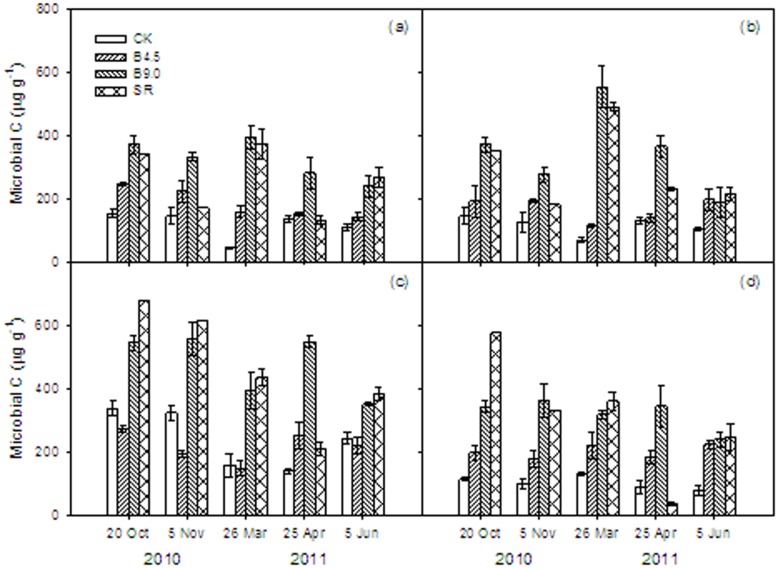
Microbial biomass carbon (MBC) under different treatments (CK: control, B4.5: 4.5 t ha^−1^ yr^−1^ biochar addition, B9.0: 9.0 t ha^−1^ yr^−1^ biochar addition, SR: incorporation of wheat straw) and different soil depths (a: 0–5, b: 5–10, c: 10–20, and d: 20–30 cm) during winter wheat season. Vertical bars represent the standard errors for means of each treatment (*n* = 3).

Variability of MBC under different rates of biochar and straw residue addition was large (Fig. 1). Treatment effects on MBC were significant (Table 1), and on average, largest in the B9.0 treatment, followed by the SR treatment, and lowest in CK. There were significant treatment*date and treatment*date*depth interactive effects (P = 0.02 and P<0.0001 respectively, Table 1). The greatest MBC was found at depth of 10–20 cm treated with SR on 20 October of 2010 (post-seeding stage), followed by the same treatment in 5 November of 2010 (before freezing stage) among all treatments and both soil layers during the whole experimental period. At depth of 0–5 and 5–10 cm, the MBC content in the treatments of B9.0 and SR in general showed the largest increases compared to CK. The MBC in the B9.0 treatment increased by 118% to 763%, while the MBC in the SR treatment was 2% lower to 722% higher compared to CK, depending on the time of year (Fig. 1a, b). The MBC at 0–5 and 5–10 cm in the B4.5 treatment also increased by 6% to 246%, depending on the time of year.

**Table 1:** Repeated Measures ANOVA P values.

Variable	Microbial C	Microbial N	Microbial C∶N
Treatm	0.0003	<0.0001	<0.0001
Date	<0.0001	<0.0001	0.002
Depth	<0.0001	0.0001	<0.0001
Treatm*date	0.02	<0.0001	0.0005
Treatm*depth	0.12	<0.0001	0.0004
Date*depth	<0.0001	0.0001	0.005
Treatm*date*depth	<0.0001	0.0003	0.0007

The MBC in the B9.0 treatment increased by 45% to 294% at soil depths of 10–20 and 20–30 cm compared to CK (Fig. 1c, d). The MBC in the SR treatment decreased by 62% during the shooting stage (25 April) at 20–30 cm soil depth, but showed some of the largest increases (between 50% and 408%) at other times at this depth. The MBC in the B4.5 treatment at 20–30 cm soil depth increased in most of the stages, but decreased at 10–20 cm soil depth by 19%, 40%, 6%, and 10% in the winter wheat post-seeding stage (20 October 2010), before freezing stage (5 November 2010), reviving stage (26 March 2011), and harvest stage (5 June 2011), respectively.

Table 2 presents the coefficients of seasonal variation of the MBC under different treatments. The CV of CK varied between 20% and 38%, and the overall CV was 25%. The overall CV of B4.5 and B9.0 treatments was 11% and 16%, respectively. The CVs were significantly smaller than that of CK. But the CV of SR treatment varied from 40% to 63%, which was significantly higher than that of CK. The CV of the surface soil tended to be higher than that of the subsoil in the same treatment, but not for the SR treatment.

**Table 2:** Coefficients of seasonal variation of MBC under different treatments (%).

Treatment	0–5 cm	5–10 cm	10–20 cm	20–30 cm	0–30 cm
CK	37.0ab	25.4ab	38.1ab	20.3b	25.0b
B4.5	25.7bc	22.9b	22.6bc	10.4c	11.2c
B9.0	19.4c	34.2ab	20.5c	15.3b	15.6c
SR	40.6ab	43.1a	40.4a	63.3a	41.6a

Note: Different small letters indicate significant differences among treatments (CK: control, B4.5: 4.5 t ha^−1^ yr^−1^ biochar addition, B9.0: 9.0 t ha^−1^ yr^−1^ biochar addition, SR: incorporation of wheat straw) at the P<0.05 level (L.S.D.).

### Microbial biomass N (MBN)

**Figure 2:**
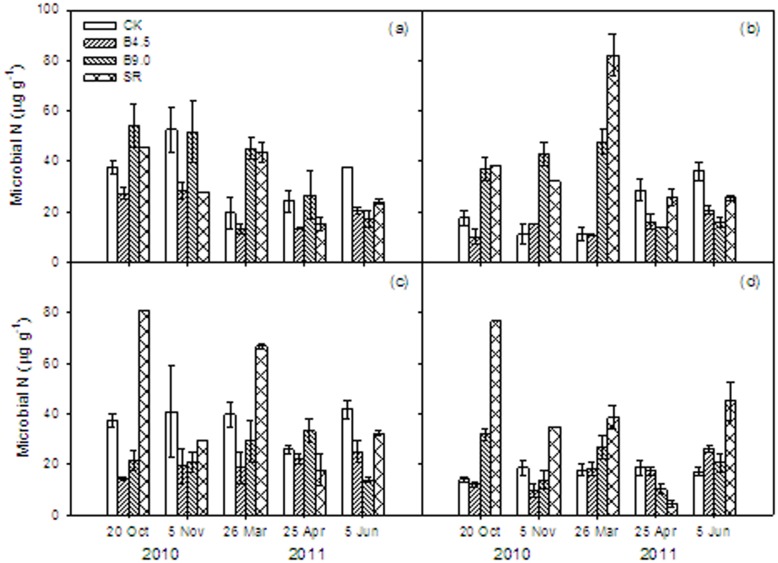
Microbial biomass nitrogen (MBN) under different treatments (CK: control, B4.5: 4.5 t ha^−1^ yr^−1^ biochar addition, B9.0: 9.0 t ha^−1^ yr^−1^ biochar addition, SR: incorporation of wheat straw) and different soil depths (a: 0–5, b: 5–10, c: 10–20, and d: 20–30 cm) during winter wheat season. Vertical bars represent the standard errors for means of each treatment (*n* = 3).

As with MBC, there was large variability in MBN among the different biochar and SR treatments, dates, and depths (Fig. 2, Table 1). However, unlike the MBC results, across date and depth the largest MBN was observed in the SR treatment (on average 35, 106, and 31% higher than the CK, B4.5 and B9.0 treatment respectively). The MBN in the B4.5 treatment decreased at most dates and soil depths, and across all dates compared to the CK treatment. Across date, the MBN in the B4.5 treatment decreased by 40, 47, 3, and 30% at 0–5, 5–10, 10–20, and 20–30 cm soil depth. The MBN in the B9.0 treatment often increased at 0–5, 5–10, and 20–30 cm soil depth during the stages of wheat post-seeding stage, before freezing stage, and reviving stage compared to the CK treatment. Large MBN pools were particularly observed in the SR treatment at 10–20 and 20–30 cm soil depth. At the shooting stage, the MBN of the four treatments were all relatively low.

The CV of MBN in the CK treatment varied from 24% to 44%, and the overall CV was 32% (Table 3). The overall CV of B4.5 was 19%, which was significantly lower than that of other treatments. The overall CV of B9.0 and SR treatments were 49% and 65% respectively, which were significantly larger than that of CK. From this, it can be seen that addition of 4.5 t ha^−1^ of biochar decreased the temporal and spatial fluctuation of MBN, but that a higher biochar addition increased the fluctuation of MBN.

**Table 3:** Coefficients of seasonal variation of MBN under different treatments (%).

Treatment	0–5 cm	5–10 cm	10–20 cm	20–30 cm	0–30 cm
CK	38.1ab	43.6ab	23.7b	31.4a	31.7b
B4.5	34.9b	23.9b	6.9c	34.9c	18.6c
B9.0	49.5a	52.5a	43.7ab	58.8b	49.1ab
SR	42.0ab	55.3a	66.9a	93.6a	65.4a

Note: Different small letters indicate significant differences among treatments (CK: control, B4.5: 4.5 t ha^−1^ yr^−1^ biochar addition, B9.0: 9.0 t ha^−1^ yr^−1^ biochar addition, SR: incorporation of wheat straw) at the P<0.05 level (L.S.D.).

### Microbial biomass C∶N ratio

**Figure 3:**
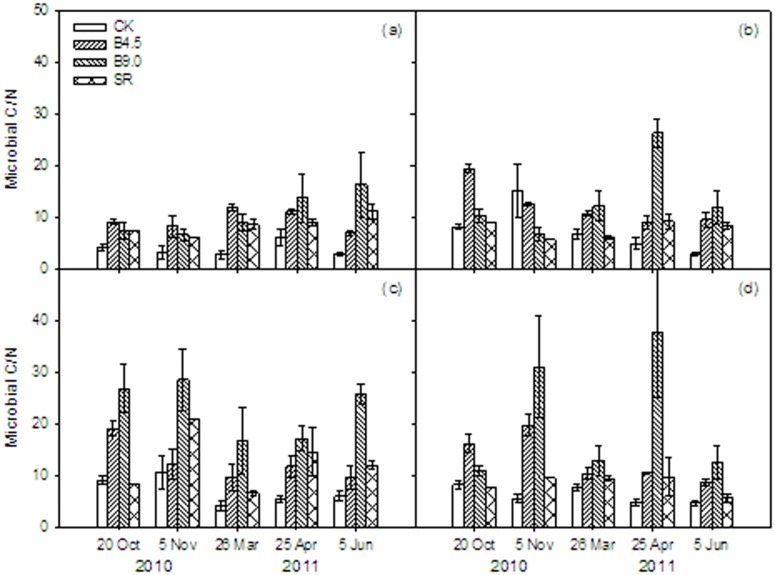
The ratio of soil microbial biomass carbon (MBC) to soil microbial biomass nitrogen (MBC/MBN) under different treatments (CK: control, B4.5: 4.5 t ha^−1^ yr^−1^ biochar addition, B9.0: 9.0 t ha^−1^ yr^−1^ biochar addition, SR: incorporation of wheat straw) and different soil depths (a: 0–5, b: 5–10, c: 10–20, and d: 20–30 cm) during winter wheat season. Vertical bars represent the standard errors for means of each treatment (*n* = 3).

Variability in microbial biomass C∶N ratio was large among the different treatments. We observed significant treatment effects on microbial biomass C∶N ratio (Table 1, Fig. 3), with largest ratios in the B9.0 treatment (on average 181% greater than CK) and B4.5 treatment (on average 93% higher than CK), suggesting that the increase in MBC with biochar addition was larger compared to the increase in MBN. The greatest microbial biomass C∶N ratio ratios were found at 10–20 cm and 20–30 cm soil depth in the B9.0 treatment.

## Discussion

### Soil microbial biomass

The change in MBC reflects the process of microbial growth, death and organic matter degradation. Our results showed that MBC increased with biochar amendment compared to CK, which suggested that microbial growth could be accelerated by biochar addition. Reported biochar effects on soil MBC are quite inconsistent. Several studies found that there was no significant effect of biochar amendment on soil MBC [8], [11]. Dempster et al. [12] found that MBC significantly decreased with biochar addition while MBN was unaltered in a coarse textured soil, and others observed the same positive effects of biochar addition on microbial biomass as ours [1], [17]–[19]. Moreover, a positive linear relationship between microbial biomass and biochar concentration was also observed in a highly weathered soil [20]. Biochar type was thought as the driving parameter for any effects on soil microbial biomass, community, and activity [9], [21].

Compared to wheat straw residue return, after 4 years of annual application, biochar increased MBC in 0–30 cm soil when applied at a high rate (9.0 t ha^−1^ yr^−1^, p<0.05), but decreased MBC when applied at a low rate (4.5 t ha^−1^ yr^−1^, p<0.01). The amount of C added in the B4.5 treatment most likely did not induce a similar increase in MBC as in the SR treatment, while the amount of C added in the B9.0 treatment did.

The MBN at 0–30 cm soil depth significantly decreased in the B4.5 treatment, but increased in the B9.0 treatment compared to CK (p<0.01), whereas the MBN at 0–30 cm soil depth in both biochar treatments were lower compared to SR treatment (p<0.05). Zavalloni et al. [8] found that biochar amendment at a rate of 5% had no significant influence on soil MBN in an incubation experiment. The increase in MBC and the decrease in MBN for biochar treatments indicate that biochar in soil acted as a carbon source rather than a nitrogen source for soil microbes. In return, this could have consequences for N cycling (e.g., increased microbial N immobilization with biochar addition). Our previous study in the field showed that biochar addition decreased soil available N by 7–10% compared to CK (p>0.05), but increased total N by 14–21% (p<0.05) in the 0–15 cm soil [22].

In the relatively unfertile and coarse-textured soil of our study, microbial biomass was probably limited by both a suitable growth environment and by C availability [17]. The labile fraction of biochar has been shown to stimulate microbial activity and abundance in some cases. Sorption of comparatively polar organic matter and nutrients could support energy for microorganisms, while macro-and micropores of biochar, which hold air and water, likely support microorganisms' livable habitat [1].Our results suggest that biochar supplied livable habitat for microorganisms stimulating microorganisms which can use the carbon source from the labile fraction of biochar.

### Soil microbial biomass C∶N

Changes in the microbial biomass C∶N ratio can reflect changes in the relative availability of C and N to microbes, but could also reflect changes in the microbial community structure. In our study, the microbial biomass C∶N ratio significantly increased with increasing biochar application and the microbial biomass C∶N ratio more than doubled in the B9.0 treatment compared to the control (Fig. 3). A pot experiment showed that there was no significant effect on the microbial biomass C∶N ratio of biochar addition at a rate of 25 t ha^−1^ [12]. Nicolardot et al. [23] determined that there was a significantly positive correlation between soil microbial biomass C∶N ratio and the C∶N ratio of the added organic matter. In contrast, Kushwaha et al. [24] reported a decrease in microbial biomass C∶N ratio after straw return, while Kallenbach and Grandy [25] found no effect on microbial biomass C∶N ratio after application of organic carbon, suggesting that changes in microbial biomass C∶N ratio cannot solely be explained by organic amendments with relatively high C content. Several studies observed a change in soil microbial structure and abundance with biochar application [1]. In our study the increased microbial C∶N ratio with biochar suggests increased microbial N limitation, while it is unclear if the increased microbial C∶N ratio was also due to changes in microbial community structure. Moreover, biochar application may alter the release of MBN or the fraction of biomass N mineralized (*K*_N_). The value of *K*_N_ had a wide range from 0.28–0.81 [26]–[28], and it was difficult to be measured precisely. The high values of microbial C∶N ratio for biochar treatments especially at 10–20 and 20–30 cm soil depths might be due to the same value of *K*_N_ used for all treatments herein. It is worthy of further study on the effect of biochar on the fraction of biomass N mineralized in the fumigation-extraction method.

### Relationship between MBC and environmental factors

**Figure 4:**
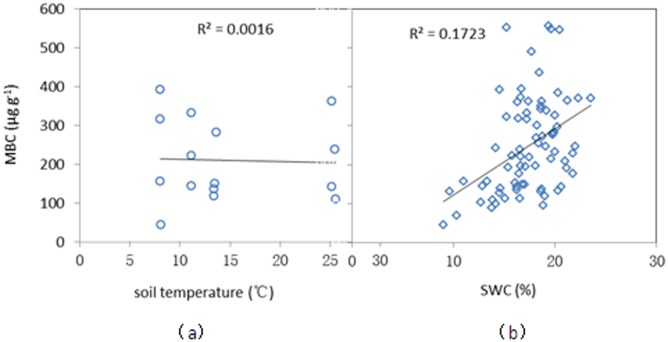
The correlations between soil microbial biomass carbon (MBC) and soil temperature (a), and between MBC and soil water content (SWC, b).

Data analysis showed that there was a significant positive correlation between SWC and MBC (**Fig. 4**, R^2^ = 0.172, P<0.001). The positive relationship between SWC and MBC suggests that SWC may have limited microbial activity at the experimental site. Biochar addition can increase soil water content [29], [30]. Therefore, biochar application could influence soil microbial biomass via variation in soil water content.

However, the increases in MBC with biochar addition did not always coincide with an increase in SWC. For instance, MBC increased sharply in the reviving stage (March 2011) in the B9.0 treatment while SWC did not. Moreover, our study showed that biochar application showed a limited effect on soil water content [13], [14], which also did not coincide with the higher MBC in the biochar treatments than in the CK treatment.

There was no significant correlation between MBC and soil temperature, and our result was supported by other studies [31], [32]. For instance, Contin et al. [32] found that MBC did not change significantly at different incubation temperatures in arable and grassland soils. But we should point out that the spike in MBC at 5–10 cm depth of soil on March 26 may be related to the soil temperature increase that occurred during the same time.

The response of microbial biomass to seasonal changes is important in regulating microbial turnover, which may in turn influence nutrient availability and ultimately plant nutrition [33]. Seasonal fluctuations in MBC in the biochar treatments were smaller than in the CK and SR treatments (Table 2), whereas, seasonal fluctuations in MBN showed no obvious differences among the CK, B9.0 and SR treatments, and only the seasonal fluctuations in the B4.5 treatment was lower than in the other treatments. Biochar may have reduced extreme environmental conditions for microbial growth, which could have important implications for C and N dynamics, and crop yield [34], [35].

## Conclusion

After 4 consecutive years of application, biochar increased soil MBC significantly compared to CK. The biochar application at a rate of 9.0 t ha^−1^ yr^−1^ reached and even exceeded the effect of wheat residue return, whereas smaller differences in soil MBN were found among treatments. Soil MBC for biochar treatments showed less seasonal fluctuation compared to CK and SR treatments, suggesting that biochar provided a more suitable habitat for soil microorganisms. Biochar treatment showed the highest value of soil microbial biomass C∶N ratio, and one possible reason might be that biochar could decease the fraction of biomass N mineralized.
